# Compensatory growth in novel Drosophila *Akt1* mutants

**DOI:** 10.1186/s13104-015-1032-0

**Published:** 2015-03-11

**Authors:** Jennifer D Slade, Brian E Staveley

**Affiliations:** Department of Biology, Memorial University of Newfoundland, 232 Elizabeth Avenue, St. John’s, Newfoundland and Labrador A1B 3X9 Canada

## Abstract

**Background:**

Organisms, tissues and cells are genetically programmed to grow to a specific largely pre-set size and shape within the appropriate developmental timing. In the event of mutation, cell death, or tissue damage, the remaining cells may increase their rate of growth to compensate and generate an intact, potentially smaller, tissue or organism in order to achieve the desired size. A delay in the developmental timing could aid in this process. The insulin receptor signalling pathway with its central component, the Akt1 kinase, and endpoint regulator, the transcription factor foxo, plays a significant role in the control of growth. *Drosophila melanogaster* is an excellent model organism with a well-studied life cycle and a consistently developing compound eye that can undergo analysis to compare changes in the properties of adult ommatidia as an indicator of growth.

**Findings:**

Imprecise excision of a *PZ* P-element inserted in the upstream region of *Akt1* generated several novel hypomorphic alleles with internally deleted regions of the Pelement. These mutations lead to small, viable Drosophila that present with delays in development. Suppression of this phenotype by the directed expression of *Akt1*^*+*^ indicates that the phenotypes observed are *Akt1* dependent. Somatic clones of the eyes, consisting of homozygous tissue in otherwise heterozygous organisms that develop within a standard timeframe, signify that more severe phenotypes are masked by an extension in the time of development of homozygous mutants. Generation of Drosophila having the hypomorphic *Akt1* alleles and a null allele of the downstream target foxo result in a phenotype very similar to that of the *foxo* mutant and do not resemble the *Akt1* mutants.

**Conclusion:**

The developmental delay of these novel *Akt1* hypomorphs results in a latent phenotype uncovered by generation of somatic clones. The compensatory growth occurring during the extended time of development appears to be implemented through alteration of foxo activity. Production of clones is an effective and informative way to observe the effects of mutations that result in small, viable, developmentally delayed flies.

## Background

The cell is the basic structural unit of all living organisms. The overall size of a cell can either augment or limit its ability to perform essential functions. Consequently size homeostasis is pertinent for the fitness and function of cells. Even slight disruption of this homeostasis can lead to disease, thus it is critical to understand the complex mechanisms that control cell growth. *Drosophila melanogaster* develops quickly through a sequence of three feeding and growing larval stages followed by pupation and eclosion [1] and is an ideal model system in which to study cell growth.

A crucial point in the control of growth in Drosophila is the achievement of the critical mass, the minimum weight required for transition from larvae to pupae, upon which any further feeding, or lack of feeding, will not prevent this change [2,3]. Drosophila larvae, when fed generously, can grow to, or past, the critical weight within four days. Restriction of dietary proteins slows this process, while total absence can halt growth completely [4]. Once larvae have reached the critical weight required for pupation, they may continue to feed for a period of time before undergoing the transition [5]. Several factors can influence the rate of growth during the larval stages including nutrition, temperature, density of organisms present in the environment, and underlying genetic mechanisms [6-10]. Slowed growth, due to genetic mechanisms or nutrient conditions, characteristically results in larvae that develop into smaller adults. While many mutations can influence growth; some alter the growth of individual organs, some retard overall growth without changing the final adult size, the mutations which slow growth and lead to a reduction in the overall organ and body size may be the most intriguing.

The conserved **in**sulin **r**eceptor (InR) signalling pathway is implicated in the management of final adult size. In Drosophila, this highly conserved pathway has been shown to control cell size and growth, and to regulate body size and nutrient usage [11,12]. When any of the seven Drosophila insulin-like peptide (Ilp) genes are overexpressed, growth rates in larvae and adults are greatly increased, and ablation of the medial neurosecretory cells in the brain (the main producer of Ilps) leads to a decrease in the growth rate and final size [13]. Overexpression of the upstream components of the pathway, including the ligand (Ilps), the insulin receptor (Inr) and the insulin receptor substrate (chico), in Drosophila results in larger than normal flies, while mutation or loss of function of these components results in size reduction and developmental delay [14]. This reinforces the pivotal role of insulin receptor signalling in the control of growth.

The Akt1 kinase is a central component of the insulin receptor signalling pathway. When Drosophila *Akt1* is overexpressed, it is shown to increase cell size but not proliferation, or number of cells, by overriding the control mechanisms that are responsible in determining the final size of cells [15]. Loss of *Akt1* can result in lethality [16] while hypomorphic activity can result in the production of smaller adults [15]. A key downstream target of Akt1, the transcription factor foxo, mediates the transcriptional regulation of the insulin pathway and controls several important cellular functions including metabolism, cell cycle regulation, DNA repair, apoptosis and protection of the cell against oxidative stress [17-20]. Through these diverse functions, the transcription factor foxo can facilitate the end result of Akt11 activity upon the regulation of cell growth and survival.

In order to explore the influence of *Akt1* activity upon cell growth, a series of novel *Akt1* hypomorphs were generated through imprecise excision of a P-element situated in the control region upstream of the gene’s coding region. A subset of these hypomorphs were selected, based upon reduction in adult size, and were further characterized with replacement analysis to confirm the reduction in size was due to altered *Akt1* activity. Due to the extended time required to reach eclosion by the homozygotes, somatic clones of the eye were generated to produce a mutant phenotype less influenced by a developmental delay. Finally, to further investigate the dynamic interaction between *Akt1* and *foxo*, Drosophila lines with novel hypomorphic alleles and a null version of the downstream target foxo gene were created and the potential for epistasis was evaluated. Our intent through these experiments is to better understand the effect of extended development time upon the overall phenotype of the novel *Akt1* hypomorphs.

## Findings

### Methods

#### Drosophila stocks, media and culture

The initial P-element insertion line *ry*^*506*^*P{PZ}Akt11*^*04226*^*/TM3, ry*^*RK*^*, Sb*^*1*^*, Ser*^*1*^ (*Akt1*^*04226*^) was obtained from the Bloomington Drosophila Stock Center. This line contains a P-element inserted within the 5′ untranslated region of the *Akt1* gene on the third chromosome. Initial reports of this allele state that it is semi-lethal [21], but we, in addition to other researchers [22,23] have found the homozygotes to be viable. The control line *w*^*1118*^*; P[FRT; w*^*+*^*]*^*2A*^*P[ry*^*+*^*neo*^*R*^*FRT]*^*82B*^*Akt1*^+^ was derived from lines obtained from Norbert Perrimon of Harvard University. The *P*∆*2-3, ry*^*+*^ line was utilized to generate the novel *Akt1* mutants [24]. The *foxo* null mutant line *w; foxo*^*W124X*^ was obtained from Drs. E. Hafen and M. Junger [25] of the University of Zurich. Wild-type Oregon R (*OrR*) stock was obtained from the Bloomington Drosophila Stock Center and *w*^*1118*^ was obtained from Dr. Howard Lipshitz from the University of Toronto. Stocks and crosses were maintained on a standard medium containing cornmeal, molasses, yeast, agar and water. Routinely, stocks were kept at room temperature (22 ± 2°C) while crosses and experiments were carried out at 25°C.

#### Generation of Drosophila lines

Hypomorphic alleles of *Akt1*^*04226*^ were generated via P-element excision by crosses to a line containing a stable source of transposase, *P*∆*2-3*. The critical class offspring of the dysgenic males and *Ly/TM3, Sb ry* females were selected based upon loss of the *PZ* P-element by the presentation of the *rosy* eye colour phenotype. These novel alleles were expected to differ from the *Akt1*^*+*^ line and *Akt1*^*04226*^ by the resultant alterations of the PZ P-element and/or the adjacent *Akt1* sequences. To allow for clonal analysis, recombinants of *w; P[FRT;w*^*+*^*]*^*2A*^*P[ry*^*+*^*neo*^*R*^*FRT]*^*82B*^ and the novel derivatives of *Akt1*^*04226*^ were generated and balanced over *TM6B, Hu Tb e*. Of the derivatives generated, a subset of these recombinants were selected for analysis based on the appearance of non-*Tubby* homozygotes.

Replacement studies were carried out by generating independent lines of *w*^*1118*^*; UAS-Akt1*^+^*/CyO; Akt1*^*m*^*/TM6B* and *w*^*1118*^*; arm-GAL4/CyO; Akt1*^*m*^*/TM6B* where *m* represents each of the novel *Akt1* mutant alleles. Crosses between these lines generated the critical class of *w*^*1118*^*; UAS-Akt1*^*+*^*/arm-GAL4; Akt1*^*m*^*/Akt1*^*m*^ to be analyzed.

The presence of FRT sites near the centromere of the 3R chromosome arm in the *Akt1*^*04226*^ derivative stocks allowed for somatic clones to be generated. The Drosophila line *y w; P{w*^*+m*^ *= GAL4-ey.H}*^*3–8*^*P{w*^*+mC*^ *= UAS-FLP1.D}*^*JD1*^*; P{ry*^*+t7.2*^ *= neoFRT}*^*82B*^*P{w*^*+mC*^ *= GMR-hid}*^*SS4*^*l*(3)*CL-R*^*1*^*/TM2* possesses *eyeless-*driven *FLP* and a distal recessive lethal allele [26] which, when crossed to each of the *Akt1*^*04226*^ derivatives generated the critical class of *y w; P{w*^*+m*^ *= GAL4-ey.H}*^*3–8*^*P{w*^*+mC*^ *= UAS-FLP1.D}*^*JD1*^*/+*; *P[FRT ;w*^*+*^*]*^*2A*^*P[ry*^*+*^*neo*^*R*^*FRT]*^*82B*^*Akt1*^*m*^*/P{ry*^*+t7.2*^ *= neoFRT}*^*82B*^*P{w*^*+mC*^ *= GMR-hid}*^*SS4*^*l*(3)*CL-R*^*1*^ where *m* represents the allele of *Akt1* derived from *Akt1*^*04226*^. The distal lethal allele resulted in the death of any homozygous *Akt1*^*+*^ cells thereby making the eye almost completely composed of homozygous *Akt1*^*m*^ cells.

Generation of flies bearing both the novel mutant *Akt1* alleles and a null *foxo* allele was performed via standard recombinant methods. As *Akt1*^*52*^ and *Akt1*^*57*^ exhibited the greater developmental delay, these alleles were selected for recombination with the null allele of *foxo*. From these combinations, a series of lines were selected based upon adult phenotypes and confirmed through PCR and sequencing.

#### Molecular characterization of the novel hypomorphs

Homozygous wild type, novel hypomorphic and double mutant Drosophila samples were collected from crosses of adult heterozygous female virgins to heterozygous males of each genotype. Ten homozygous male flies were collected upon eclosion and aged three to five days before being flash-frozen at −70°C. DNA was extracted from each sample via a standard phenol-chloroform protocol. The Flybase database (http://flybase.org) includes the complete sequence for the PZ P-element positioned within the *Akt1*^*04226*^ line, and the National Center for Biotechnology Information (NCBI) database (http://www.ncbi.nlm.nih.gov/) includes the complete gene sequence for *Akt1*^*+*^. To design oligonucleotides indicating the breakpoint region of each novel mutant, a series of oligonucleotides both flanking and spanning the P-element insertion site was carried out via Primer3. PCR analysis via primers spanning the PZ Pelement revealed forward primer sites present and positioned near the breakpoints. A reverse primer positioned near the end of the PZ P-element allowed for generation of breakpoint fragments using HotStart Taq Polymerase (Qiagen Inc.) in an Eppendorf Mastercycler gradient thermal cycler through standard methods. Gels were photographed with a ChemiImageTM Ready 4400 v5.5 photo-documentation system. Purification and sequencing of the PCR products was completed at the Genomics and Proteomics (GaP) facility, Memorial University of Newfoundland.

#### Analysis of developmental timing

Heterozygotes are identified based upon the presence of *Humeral (Hu)*, an allele of *Antennapedia* carried by the *TM6B, Hu Tb e* balancer chromosome that results in extra bristles on the outer edges of the prothorax, while homozygous mutant flies lack this marker. Heterozygous females and males of the novel *Akt1* hypomorphs were transferred to fresh media, incubated at 25°C for six hours to allow for egg-laying then removed. Vials were returned to the incubator immediately after removal of adult flies and examined each morning for fifteen days. Observations included days until pupation and eclosion. Pupae and adult flies were scored as *Tubby* heterozygotes or non-*Tubby* mutants and used to generate developmental delay line graphs in GraphPad Prism Version 5.03.

#### Biometric analysis of Drosophila eyes

Critical class males of the homozygous mutant *Akt1* alleles, the transgenic rescues, the somatic clones and the double mutant lines were collected and aged for three days. Flies were then flash-frozen at −70°C before preparation for scanning electron microscopy. Preparation included mounting upon aluminum SEM studs, desiccated and sputter coating in gold. Images were taken with either a Hitachi S-170 or S-570 Scanning Electron Microscope as per standard methods and analyzed using NIH Image J software [27].

### Results

#### Three novel Akt1 mutants retain portions of inserted PZ P-element

**Figure 1:**
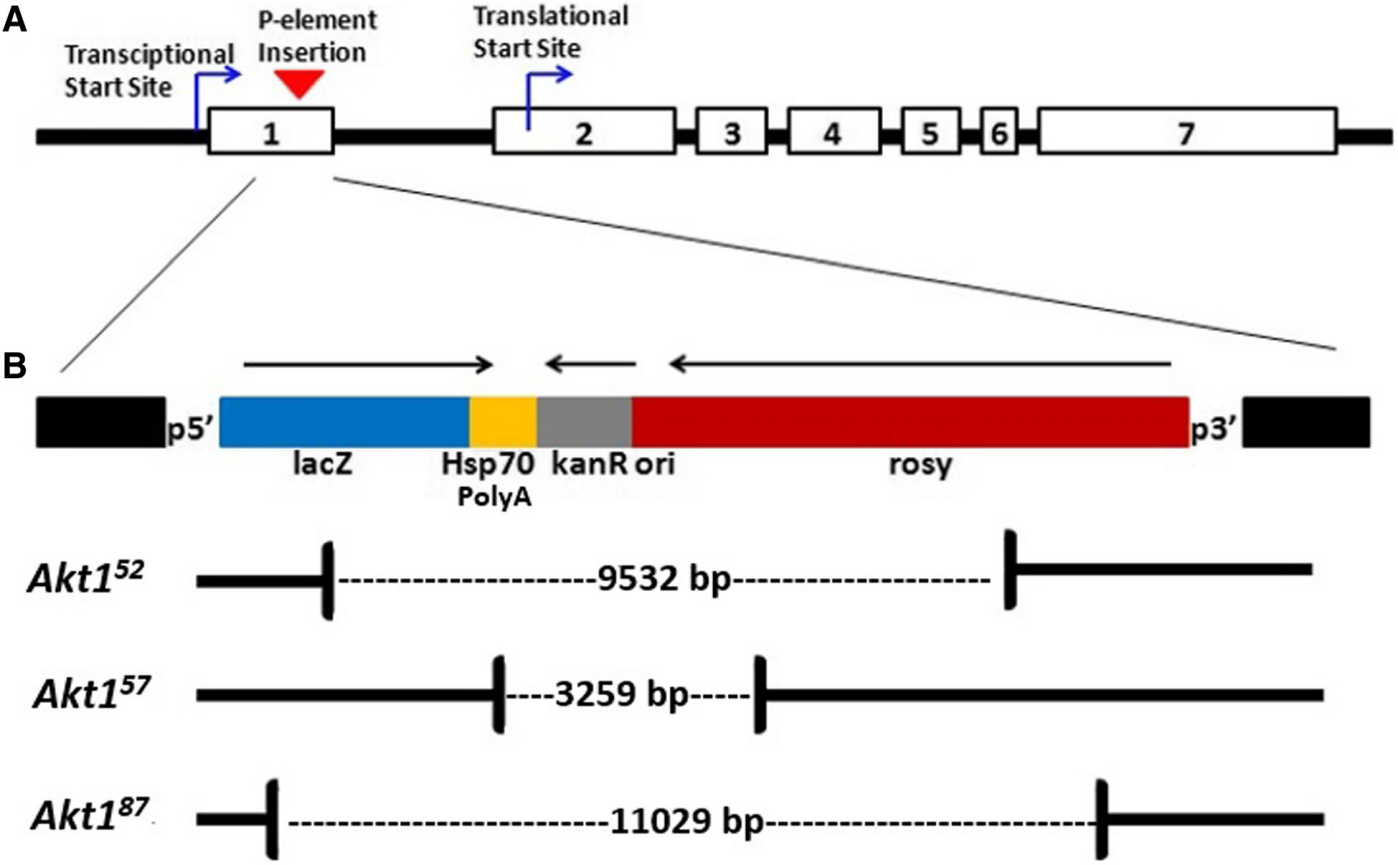
**Novel hypomorphic alleles of** ***Akt1*** ^***+***^ **retain PZ P-element sequences within exon 1. A)** Gene map of *Akt1* ^*+*^, transcript variant B (Genbank accession: NM_169707.2). White boxes represent each of the seven exons. The P-element insertion site is located within exon 1 between the transcriptional and translational start sites. **B)** Molecular characterization confirms the base pair sizes of the internally deleted sections for each hypomorphic allele. Analysis indicates that each allele retains various portions of both the *lacZ* and *rosy* genes located either end of the PZ P-element.

Molecular characterization of the three small viable *Akt1* mutants revealed internally deleted versions of the *PZ* P-element at the original point of insertion (Figure 1). Analysis indicates that the retained sections are from both ends of the *PZ* P-element. *Akt1*^*87*^ possesses the largest deleted region (11029 base pairs) between nucleic acids 2184 (within *lacZ*) and 13213 (within *ry*^*+*^) of the *PZ* P-element sequence. The next largest deletion (9532 base pairs) is in *Akt1*^*52*^ between nucleic acids 2754 (within *lacZ*) and 12307 (within *ry*^*+*^). Lastly *Akt1*^*57*^ has the smallest deletion of the three mutants of 3259 base pairs between nucleic acid 4315 (within the *HSP70* poly-adenylation control region) and 7574 (within *ry*^*+*^). Each deletion includes a part of the *ry*^*+*^ gene, responsible for the phenotype (*ry*^*−*^) upon which these mutants were selected. No alteration to the *Akt1* coding region sequence was detected in the three mutants.

#### Three novel Akt1 mutants are developmentally delayed

**Figure 2:**
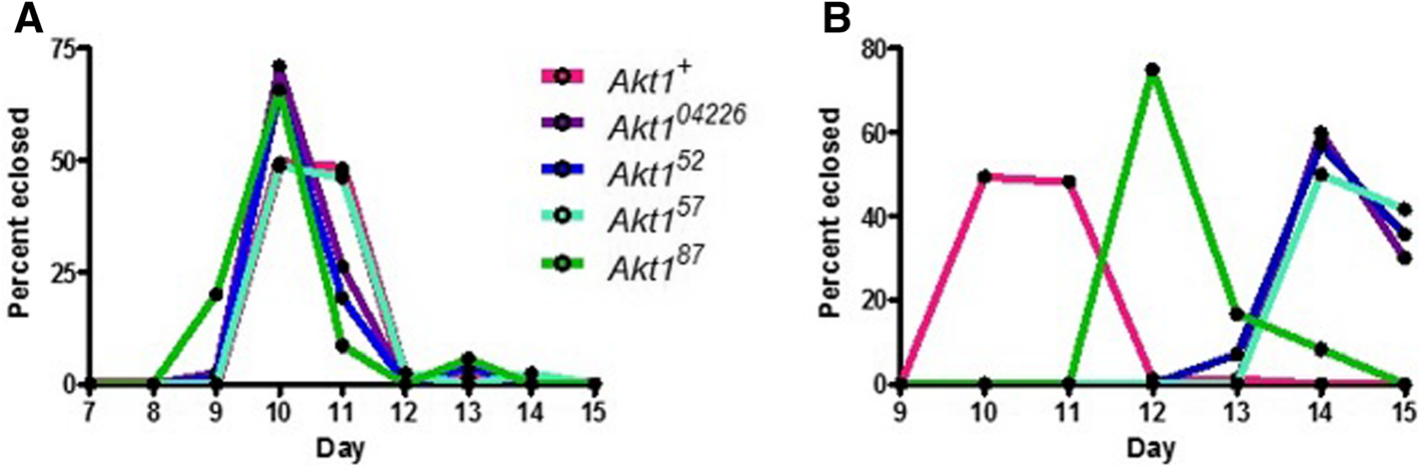
**Novel** ***Akt1*** **mutants are developmentally delayed.** Data of developmental delay experiment was plotted as percent eclosed versus day as a line graph. This method allows the peaks of each line to clearly represent the day of which most adult flies eclosed for each genotype. N = 85 (*Akt1* ^*+*^), 48 (*Akt1* ^*04226*^), 40 (*Akt1* ^*52*^), 53 (*Akt1* ^*57*^) and 47 (*Akt1* ^*87*^). **A)** Heterozygotes eclose by day ten along with the control. **B)** The homozygous mutants are delayed in growth and do not eclose until two to four days later than the controls.

In the development from embryo to adult, *Akt1* mutant heterozygotes are similar to controls (Figure 2). The formation of heterozygous (*Tubby*) pupae occurs in a similar time-frame to the control lines (data not shown). The time required to eclose by adult *Akt1* heterozygotes (*Humeral*) and control flies is nine to ten days (Figure 2). Emergence of the homozygous adult flies is delayed by two to four days. One allele, *Akt1*^*87*^, is delayed until day 12 while the other alleles are delayed until day 14 (Figure 2). The extended time of development of homozygotes may be required for the production of the adult mutants.

#### The eyes of novel Akt1 mutants are reduced in ommatidia size and number

**Figure 3:**
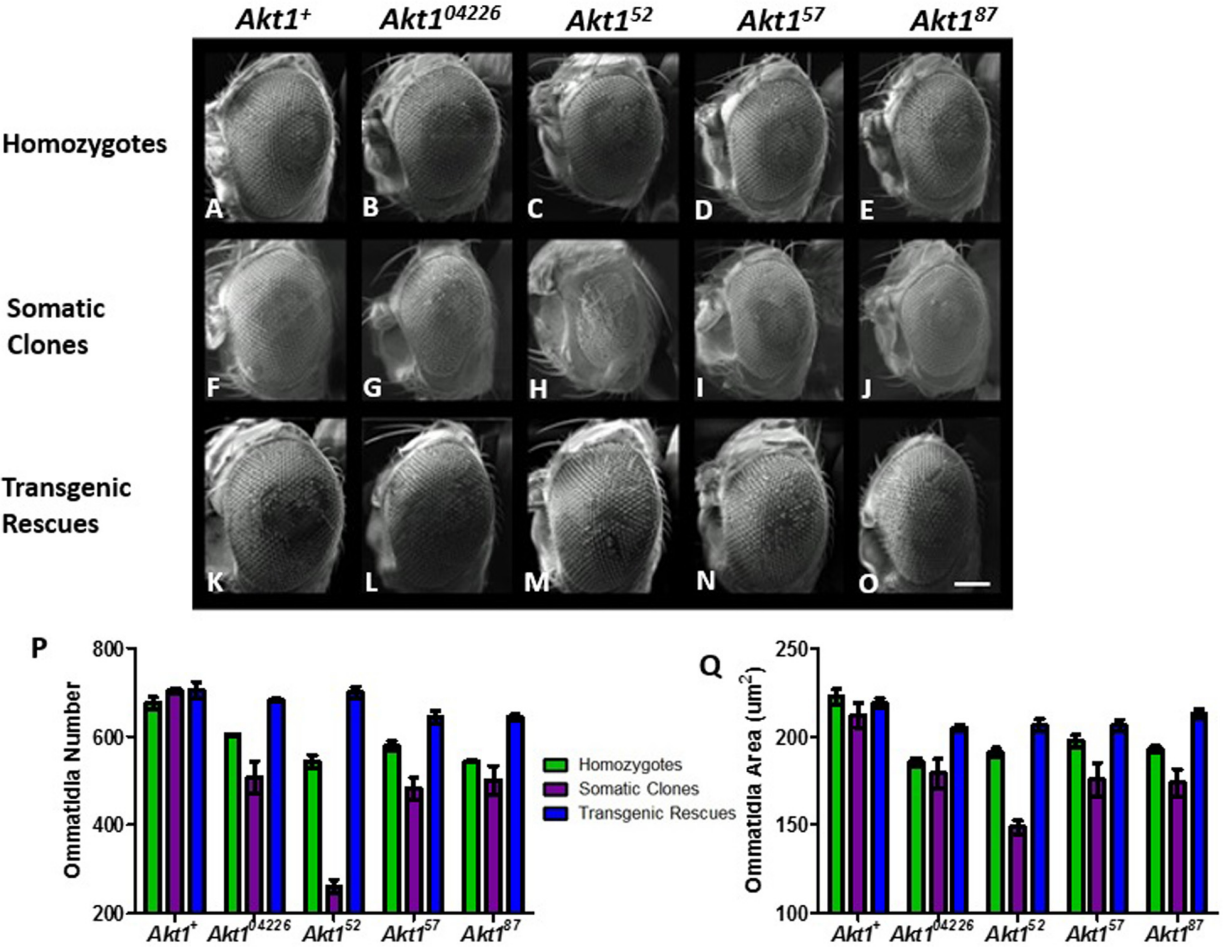
**Novel** ***Akt1*** **mutants are reduced in both ommatidia size and number, enhanced in somatic clones and partially rescued by expression of** ***Akt1*** ^***+***^ **.** Scanning electron micrographs illustrate the phenotypes of the homozygote, somatic clone and transgenic rescue of the control, original P-element insertion mutant and each of the three novel *Akt1* mutants. Top row images **(A-E)** of homozygous mutants signify that hypomorphic alleles of *Akt1* are reduced in size. The middle row images **(F-J)** of somatic clones of these alleles reveal an increase in severity of this phenotype. The bottom row of images **(K-O)** express wild type *Akt1* in the background of the hypomorphic alleles and validate these phenotypes are *Akt1* dependent. Full genotypes are **A**: *w; P[FRT;w* ^*+*^ *]* ^*2A*^ *P[ry* ^*+*^ *neo* ^*R*^ *FRT]* ^*82B*^ *Akt1* ^*+*^ */Akt1* ^*+*^ **B**: *w; P[FRT;w* ^*+*^ *]* ^*2A*^ *P[ry* ^*+*^ *neo* ^*R*^ *FRT]* ^*82B.*^ *Akt1* ^*04226*^ */Akt1* ^*04226*^ **C**: *w; P[FRT;w* ^*+*^ *]* ^*2A*^ *P[ry* ^*+*^ *neo* ^*R*^ *FRT]* ^*82B*^ *Akt1* ^*52*^ */Akt1* ^*52*^ **D**: *w; P[FRT;w* ^*+*^ *]* ^*2A*^ *P[ry* ^*+*^ *neo* ^*R*^ *FRT]* ^*82B*^ *Akt1* ^*57*^ */Akt1* ^*57*^ **E**: *w; P[FRT; w* ^*+*^ *]* ^*2A*^ *P[ry* ^*+*^ *neo* ^*R*^ *FRT]* ^*82B*^ *Akt1* ^*87*^ */Akt1* ^*87*^ **F**: *y w; P{w* ^*+m*^ *= GAL4-ey.H}* ^*3–8*^ *P{w* ^*+mC*^ *= UAS-FLP1.D}* ^*JD1*^ */+*; *P[FRT ;w* ^*+*^ *]* ^*2A*^ *P[ry* ^*+*^ *neo* ^*R*^ *FRT]* ^*82B*^ *Akt1* ^*+*^ */P{ry* ^*+t7.2*^ *= neoFRT}* ^*82B*^ *P{w* ^*+mC*^ *= GMR-hid}* ^*SS4*^ *l*(3)*CL-R* ^*1*^ **G**: *y w; P{w* ^*+m*^ *= GAL4-ey.H}* ^*3–8*^ *P{w* ^*+mC*^ *= UAS-FLP1.D}* ^*JD1*^ */+*; *P[FRT; w* ^*+*^ *]* ^*2A*^ *P[ry* ^*+*^ *neo* ^*R*^ *FRT]82B Akt104226/ P{ry + t7.2 = neoFRT}82B P{w + mC = GMR-hid}SS4 l*(3)*CL-R1* **H**: *y w; P{w* ^*+m*^ *= GAL4-ey.H}* ^*3–8*^ *P{w* ^*+mC*^ *= UAS-FLP1.D}* ^*JD1*^ */+*; *P[FRT;w* ^*+*^ *]* ^*2A*^ *P[ry* ^*+*^ *neo* ^*R*^ *FRT]* ^*82B*^ *Akt1* ^*52*^ */P{ry* ^*+t7.2*^ *= neoFRT}* ^*82B*^ *P{w* ^*+mC*^ *= GMR-hid}* ^*SS4*^ *l*(3)*CL-R* ^*1*^ **I**: *y w; P{w* ^*+m*^ *= GAL4ey.H}* ^*3–8*^ *P{w* ^*+mC*^ *= UAS-FLP1.D}* ^*JD1*^ */+*; *P[FRT ;w* ^*+*^ *]* ^*2A*^ *P[ry* ^*+*^ *neo* ^*R*^ *FRT]* ^*82B*^ *Akt1* ^*57*^ */P{ry* ^*+t7.2*^ *= neoFRT}* ^*82B*^ *P{w* ^*+mC*^ *= GMR-hid}* ^*SS4*^ *l*(3)*CL-R* ^*1*^ **J**: *y w; P{w* ^*+m*^ *= GAL4-ey.H}* ^*3–8*^ *P{w* ^*+mC*^ *= UAS-FLP1.D}* ^*JD1*^ */+*; *P[FRT ;w* ^*+*^ *]* ^*2A*^ *P[ry* ^*+*^ *neo* ^*R*^ *FRT]* ^*82B*^ *Akt1* ^*87*^ */*P{ry^+t7.2^ = neoFRT}^82B^ P{w^+mC^ = GMR-hid}^SS4^ l(3)CL-R^1^ ***K***: w^1118^; UAS-Akt1^+^/arm-GAL4; *Akt1* ^*+*^ */Akt1* ^*+*^ **L**: *w* ^*1118*^ *; UAS-Akt1* ^*+*^ */arm-GAL4; Akt1* ^*04226*^ */Akt1* ^*04226*^ **M**: *w* ^*1118*^ *; UASAkt1* ^*+*^ */arm-GAL4; Akt1* ^*52*^ */Akt1* ^*52*^ **N**: *w* ^*1118*^ *; UAS-Akt1* ^*+*^ */arm-GAL4; Akt1* ^*57*^ */Akt1* ^*57*^ **O**: *w* ^*1118*^ *; UAS-Akt1* ^*+*^ */arm-GAL4; Akt1* ^*87*^ */Akt1* ^*87*^ *.* Scale bar = 100 um. Biometric analysis of both the number, **(P)** and area, **(Q)** of ommatidia quantifies the subtlety of these size differences. N values can be found in Table 1. Green bars represent the homozygotes; purple bars represent the clones and blue bars represent the transgenic rescues. The original P-element insertion mutant *Akt1* ^*04226*^ is smaller than the control but larger than the novel mutants in both size and number. All three novel *Akt1* mutants are reduced in both the area and number of ommatidia as both homozygotes or clones but reach almost comparable measurements compared to the control when rescued by a transgene. Error bars represent standard error of the mean (p = <0.05). Further statistical analysis can be found in Table 1.

Biometric analysis of homozygous *Akt1* mutant eyes indicates there is an overall decrease in both ommatidia number and size when compared to controls (Table 1, Figure 3). The control eyes had a number of 676.4 ± 13.2 ommatidia per eye (OPE) and an ommatidia area of 222.6 (±4.3) um^2^, which was the largest overall. The original Pelement insertion mutant *Akt1*^*04226*^ has the smallest ommatidia area of 185.6 ± 2.4 um^2^, but with an ommatidia number of 604 ± 2 OPE. The three novel *Akt1* hypomorphs were all smaller than the control in ommatidia area and significantly reduced in ommatidia number when compared to both the control and the original P-element insertion mutant. Of these three, *Akt1*^*52*^ is the smallest with a count of 544.8 ± 14.1 OPE and an area of 191.1 ± 2.4 um^2^. The mutant *Akt1*^*87*^ is slightly larger with an ommatidia area of 192.8 ± 2.1 um^2^ and 545.4 ± 2.5 OPE. The largest of the mutants is *Akt1*^*57*^ with an ommatidia area of 197.6 ± 3.5 um^2^ and an ommatidia number of 579.4 ± 11 OPE.

**Table 1:** **Biometric analysis of ommatidia area and number in homozygous mutant, transgenic rescue and somatic clones of novel** ***Akt1*** **mutant alleles**

**Allele**	**a) Homozygotes**									
	**N**	**OA**	**P1**	**P2**	**P3**	**N**	**ON**	**P1**	**P2**	**P3**
***Akt1*** ^***+***^	15	222.6 ± 4.3	N/A	0.5286	<0.0001	5	676.4 ± 13.2	N/A	0.2179	0.1671
				NS	S				NS	NS
***Akt1*** ^***04226***^	15	185.6 ± 2.4	<0.0001	<0.0001	<0.0001	5	604 ± 2	0.0006	<0.0001	0.0007
			S	S	S			S	S	S
***Akt1*** ^***52***^	15	191.1 ± 2.4	<0.0001	0.0019	<0.0001	5	544.8 ± 14.1	0.0001	<0.0001	<0.0001
			S	S	S			S	S	S
***Akt1*** ^***57***^	15	197.6 ± 3.5	0.0002	0.0741	<0.0001	5	579.4 ± 11	0.0005	0.0057	0.0017
			S	NS	S			S	S	S
***Akt1*** ^***87***^	15	192.8 ± 2.1	<0.0001	<0.0001	<0.0001	5	545.4 ± 2.5	<0.0001	<0.0001	0.0214
			S	S	S			S	S	S
**Allele**	**b) Transgenic Rescues**									
	**N**	**OA**	**P1**			**N**	**ON**	**P1**		
***Akt1*** ^***+***^	15	218.9 ± 2.7	N/A			5	705.8 ± 17.6	N/A		
***Akt1*** ^***04226***^	15	204.7 ± 1.8	0.0002			5	683.2 ± 3.9	0.2445		
			S					NS		
***Akt1*** ^***52***^	15	206.3 ± 3.6	0.0086			5	701 ± 12.6	0.8297		
			S					NS		
***Akt1*** ^***57***^	15	206.2 ± 3.2	0.0052			5	644.6 ± 13.5	0.0246		
			S					S		
***Akt1*** ^***87***^	15	212.9 ± 2.5	0.1112			5	643.6 ± 7.1	0.0112		
			NS					S		
**Allele**	**c) Somatic Clones**									
	**N**	**OA**	**P1**			**N**	**ON**	**P1**		
***Akt1*** ^***+***^	9	212.1 ± 7.1	N/A			3	704.3 ± 5.5	N/A		
***Akt1*** ^***04226***^	9	179.3 ± 8.3	<0.0001			3	508.3 ± 35.4	0.0007		
			S					S		
***Akt1*** ^***52***^	9	148.7 ± 3.9	<0.0001			3	261 ± 14.3	<0.0001		
			S					S		
***Akt1*** ^***57***^	9	175.7 ± 9.6	<0.0001			3	483 ± 24.5	0.0001		
			S					S		
***Akt1*** ^***87***^	9	173.8 ± 7.9	<0.0001			3	501 ± 33.2	0.0005		
			S					S		

OA = Ommatidia Area (um^2^), ON = Ommatidia Number, P1 = P-value when compared to *Akt1* ^*+*^ control, P2 = P-value when compared to transgenic rescue counterpart, P3 = P-value when compared to somatic clone counterpart, S = significant, NS = not significant.

#### Transgenic replacement partially rescue the phenotype of mutant homozygotes

Ubiquitous expression of wild-type *Akt1* in the background of the homozygous mutants results in a partial rescue of both ommatidia size and number (Table 1, Figure 3). The transgenic control *Akt1*^*+*^ is the largest eye overall having an ommatidia area of 218.9 ± 2.7 um^2^ and a total number of 705.8 ± 17.6 OPE. The transgenic expression of *Akt1*^*+*^ in the background of the original P-element insertion mutant results in eyes that are only slightly smaller than the control having an ommatidia area of 204.7 ± 1.8 um^2^ and a total of 683.2 ± 3.9 OPE. In all cases the size of the ommatidia and the total count of ommatidia for the mutants with transgenic replacement of wild-type *Akt1*^*+*^ does not differ significantly from the control (Figure 3). The average ommatidia area is very similar for *Akt1*^*52*^ and *Akt1*^*57*^ being 206.3 ± 3.6 um^2^ and 206.2 ± 3.2 um^2^ respectively; while the area for *Akt1*^*87*^ is larger at 212.9 ± 2.5 um^2^. The ommatidia number for the partially rescued mutants is similar for *Akt1*^*57*^ and *Akt1*^*87*^ being 644.6 ± 13.5 OPE and 643.6 ± 7.1 OPE respectively, with *Akt1*^*52*^ having a few more ommatidia at 701 ± 12.6 OPE.

#### Somatic clones of the eye have a more severe phenotype than the homozygotes

Given the developmental delay of the *Akt1* homozygotes, analysis of somatic clones of the eye was carried out. The FLP recombinase was driven by the *eyeless* promoter to direct expression in the developing eye tissue. In the presence of FLP, homologous chromosomes undergo mitotic recombination between the FRT sites located on chromosome pairs. Heterozygous parent cells can produce both homozygous *Akt1* mutant cells containing two copies of the mutant allele, and cells containing two copies of *Akt1*^*+*^. In this system, the *Akt1*^*+*^ daughter cells are lost due to the presence of a linked recessive cell lethal mutation located on the same arm of the chromosome bearing the *Akt1*^*+*^ allele. Thus, in the eyes of clone bearing flies, the surviving cells bear two copies of the *Akt1* mutant allele under investigation. The clone of the control is the largest and most consistent in size when compared to its homozygous and transgenic rescued counterpart having an ommatidia area of 212.1 ± 7.1 um^2^ and number of 704.3 ± 5.5 OPE. The cloned original P-element insertion mutant *Akt1*^*04226*^ is reduced in both size and number compared to its original homozygous version with an average area of 179.3 ± 8.3 um^2^ and ommatidia number of 508.3 ± 35.4 OPE, yet is comparable in size and number to two of the cloned novel mutants, *Akt1*^*57*^ and *Akt1*^*87*^, which have an ommatidia area of 175.7 ± 9.6 um^2^ and 173.8 ± 7.9 um^2^ and a count of 483 ± 24.5 OPE and 501 ± 33.2 OPE respectively. The measurement and count of ommatidia for both of these mutants is significantly smaller than that of their homozygous versions. Of all the mutants, *Akt1*^*52*^ exhibits the most severe phenotype with the greatest decrease in ommatidia area (148.7 ± 3.9 um^2^) and number (261 ± 14.3 OPE) when compared to both its homozygous counterpart as well as with the other cloned mutants.

#### Akt1-foxo double mutant lines reveal an epistatic relationship

**Figure 4:**
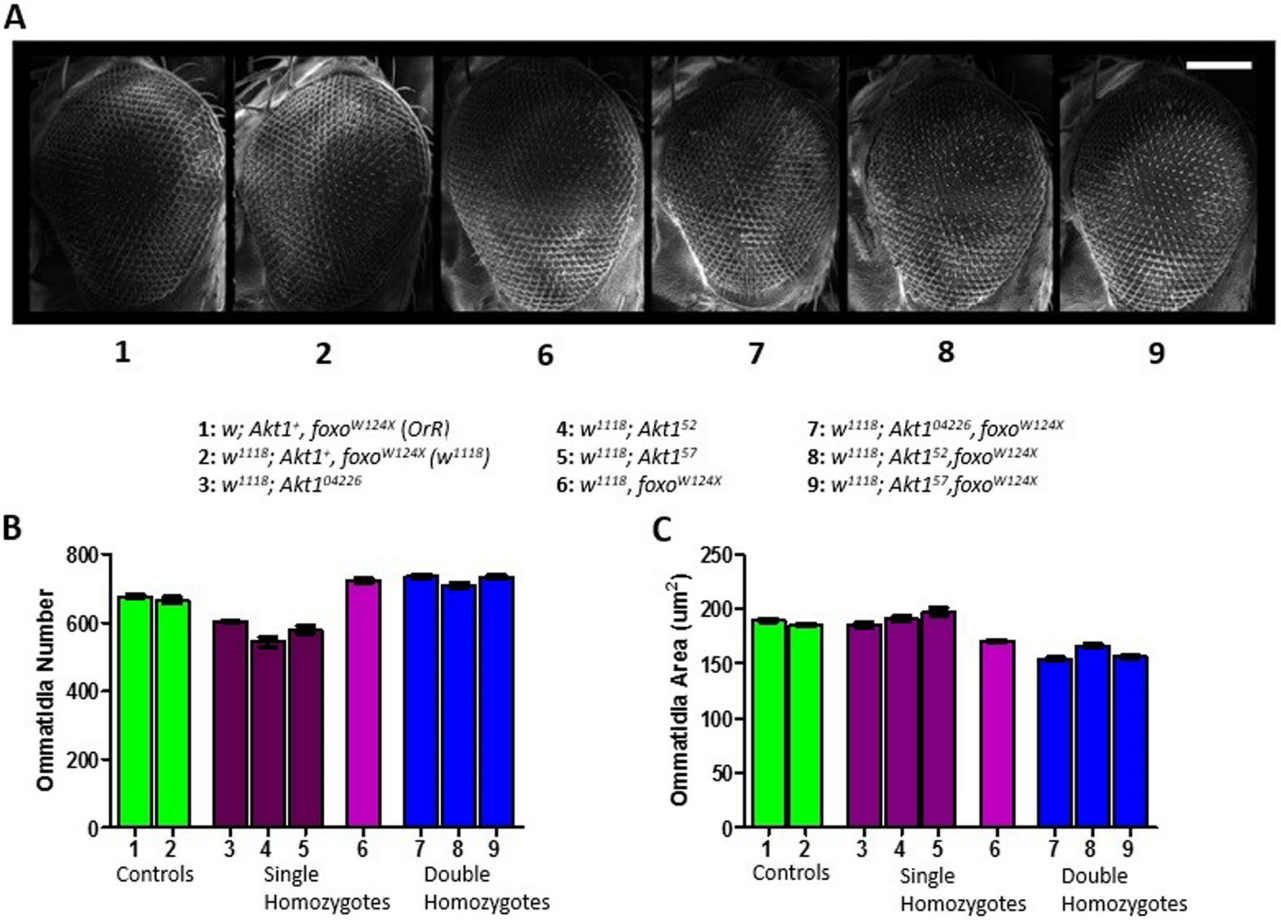
**Double** ***Akt1/foxo*** **mutants demonstrate an epistatic effect upon growth. A**. Scanning electron micrographs of Drosophila eyes bearing hypomorphic alleles of *Akt1* and null mutations of the *foxo* gene indicates that the double mutants more closely resemble the phenotype of the null *foxo* mutation. Representative images of genotypes 3, 4 and 5 can be found in Figure 3. Scale bar = 100 um. Biometric analysis quantifies this similarity in terms of ommatidia number **(B)** and size **(C)**. N values can be found in Table 2. Green bars represent the controls, purple bars represent the novel *Akt1* mutants and the null *foxo* mutant, blue bars represent the double mutant homozygotes. In analysis of both the ommatidia size and number, the double mutants have a larger number of smaller ommatidia in comparison to the original *Akt1* mutants, but are comparable in both size and number to the null *foxo* mutant. Error bars represent standard error of the mean (p = <0.05). Further statistical analysis can be found in Table 2.

Drosophila lines having both the novel *Akt1* mutant alleles in combination with a null *foxo* mutant allele resemble the original *foxo* mutant more closely than the *Akt1* mutants (Figure 4; Table 2). The controls *OrR* and *w*^*1118*^ have an ommatidia area of 189.4 ± 1.43 um^2^ and 185.5 ± 1.34 um^2^ and an ommatidia count of 675.7 ± 4.4 OPE and 665.3 ± 9.6 OPE respectively. In comparison, the null *foxo* mutant eye has a smaller average ommatidia area of 170.4 ± 1.42 um^2^ and a higher ommatidia count of 723 ± 5.6 OPE. The double mutant lines are smaller than both the controls and the null *foxo* mutant in size of ommatidia, but have counts of ommatidia that are not significantly different from the null *foxo* mutant. Both the double mutants bearing *Akt1*^*04226*^ and *Akt1*^*57*^ alleles have similar ommatidia areas of 154.4 ± 1.2 um^2^ and 156.3 ± 1.1 um^2^, and ommatidia numbers of 735 ± 5.2 OPE and 733.9 ± 6.3 OPE respectively. The double mutant bearing the *Akt1*^*52*^ allele is closer to the null *foxo* mutant in ommatidia size (166.3 ± 1.7 um^2^) but has slightly fewer ommatidia with 709.1 ± 8.2 OPE. The ommatidia size for each of the double mutants is considerably smaller than the original homozygous mutant versions of each *Akt1* mutant allele, while the counts of ommatidia are much higher, exhibiting the same trend as the null *foxo* mutant in comparison to the novel *Akt1* mutants.

**Table 2:** **Biometric analysis of ommatida area and number of Drosophila eyes bearing both a novel** ***Akt1*** **mutant allele and a null** ***foxo*** **mutant allele**

**Genotype**	**N**	**OA**	**P1**	**P2**	**P3**	**P4**	**N**	**ON**	**P1**	**P2**	**P3**	**P4**
***w*** ^***+***^ ***; Akt1*** ^***+***^ ***foxo*** ^***+***^ ***(OrR)***	48	189.4 ± 1.4	N/A	N/A	N/A	N/A	16	675.7 ± 4.4	N/A	N/A	N/A	N/A
***w*** ^***1118***^ ***; Akt1*** ^***+***^ ***foxo*** ^***+***^ ***(w*** ^***1118***^ ***)***	36	185.5 ± 1.3	N/A	N/A	N/A	N/A	12	665.3 ± 9.6	N/A	N/A	N/A	N/A
***w*** ^***1118***^ ***; Akt1*** ^***+***^ ***foxo*** ^***W124X***^	42	170.4 ± 1.4	<0.0001	<0.0001	N/A	N/A	14	723.0 ± 5.6	<0.0001	<0.0001	N/A	N/A
			S	S					S	S		
***w*** ^***1118***^ ***; Akt1*** ^***04226***^ ***foxo*** ^***W124X***^	45	154.4 ± 1.2	<0.0001	<0.0001	<0.0001	<0.0001	15	735.0 ± 5.2	<0.0001	<0.0001	<0.0001	0.1282
			S	S	S	S			S	S	S	NS
***w*** ^***1118***^ ***; Akt1*** ^***52***^ ***foxo*** ^***W124X***^	39	166.3 ± 1.7	<0.0001	<0.0001	<0.0001	0.0630	13	709.1 ± 8.2	0.0008	0.0020	<0.0001	0.1686
			S	S	S	NS			S	S	S	NS
***w*** ^***1118***^ ***; Akt1*** ^***57***^ ***foxo*** ^***W124X***^	54	156.3 ± 1.1	<0.0001	<0.0001	<0.0001	<0.0001	18	733.9 ± 6.3	<0.0001	<0.0001	<0.0001	0.2212
			S	S	S	S			S	S	S	NS

OA = Ommatidia Area (um^2^), ON = Ommatidia Number, P1 = P-value when compared to the *Oregon R (OrR)* control, P2 = P-value when compared to the *w* ^*1118*^ control, P3 = P-value when compared to the mutant *Akt1* homozygote counterpart, P4 = P-value when compared to *w* ^*1118*^ *; Akt1* ^*+*^ *foxo* ^*W124X*^, S = significant, NS = not significant.

### Discussion

Viable novel *Akt1* hypomorphs were generated via imprecise P-element excision and were found to retain internally deleted versions of the original *PZ* P-element upstream of the *Akt1* gene’s protein coding region (Figure 1). Three selected *Akt1* hypomorphs were characterized phenotypically as small in size and delayed in terms of developmental time. In Drosophila, the development from egg to adult involves three larval stages plus pupation before the non-growing sexually mature adult fly arises. The timing of transition between these stages is dependent upon the rate of growth. The insulin receptor signalling pathway is a major contributor in the control of growth and has been implicated in the control of the onset of metamorphosis in Drosophila [28]. Ablation of insulin producing cells within the larval brain decreases the growth rate and delays metamorphosis in Drosophila, as does a loss-of-function mutation of the insulin receptor [29,30]. As Akt1 is a central component of the insulin receptor signalling pathway, it is not surprising that these novel hypomorphic alleles result in a delay of development and overall smaller adult organisms.

Due to the extension in the time for the novel mutants to undergo eclosion, a comparison of the phenotypes of the eye for both homozygous mutants and somatic clones was undertaken. The clone eyes are comprised of homozygous mutant tissue in a heterozygous organism that develops within a relatively normal timeframe. Biometric analysis of the eyes of these mutant clones revealed enhanced severity of the decreased growth phenotype. Adult organisms, as well as their organs and tissues, have a tendency to develop within a range of normal overall size, such that the cellular composition may vary from a large number of small cells, to a small number of large cells. Cell growth includes an increase in cell number and cell size, and while not mutually exclusive, both can be regulated by distinct extracellular processes [11,31-33], including the insulin receptor signalling pathway, which is highly conserved between invertebrates and mammals [34]. Reduced expression or loss of *Akt1,* the central component of insulin receptor signalling, can result in the production of smaller animals or, if severe, lethality [35,16,15]. The smaller eyes observed in the homozygotes is expected with the lower expression of *Akt1* in these novel mutants.

Compensatory growth is widespread and occurs in the surviving cells of damaged tissues to generate final structures of near normal overall size [36,37]. This growth consists of remodeling the existing tissue to regenerate the full body plan in response to tissue damage leading to the development of a smaller but still complete and intact organism. In order to maintain tissue homeostasis, cells that survive the tissue damage can compensate for those that are lost by increasing their rate of proliferation and cell divisions. Cells in Drosophila that have experienced an increase in cell death via radiation showed an increase in proliferation by the surviving cells [38]. Compensatory proliferation has been shown to lead to the development of normal-sized adult wings even when 40-60% of cells in the wing disc of Drosophila are either killed or rendered incapable of further proliferation [39]. These novel *Akt1* hypomorphs have been shown to be developmentally delayed and result in the formation of small adult flies. The mutant clone eyes show a more severe phenotype due to the reduced replacement of missing tissue without the extended time during development. Clearly, the extended period of time required for these mutants to develop allows compensatory proliferation to generate smaller but intact adults.

In order to begin to understand the mechanisms responsible for the observed compensatory growth, double mutant lines of the novel *Akt1* hypomorphs and an amorphic allele of *foxo*, a gene encoding a key downstream target of *Akt1*, were generated. The transcription factor foxo is known as a major effector of insulin receptor signalling and has been implicated in the control of cell growth. Overexpression of the mammalian homologues of *foxo*, as well as Drosophila *foxo*, leads to growth arrest [7,40] which can be suppressed with increased insulin receptor signalling. This suppression is ineffective when the foxo transcription factor has been made incapable of phosphorylation, and thus nuclear exclusion, by Akt1. In addition to this, *foxo* governs the expression of target genes that encode factors that regulate cell growth such as the eukaryotic initiation factor 4E-binding protein (4E-BP) gene and cell cycle regulators including p27^kip1^. The *4E-BP* product is a negative regulator of protein synthesis and has been shown to strongly influence the regulation of cell growth [41]. When foxo is upregulated, so is 4E-BP, which binds to the messenger RNA 5′ cap-binding protein eIF4E to inhibit protein synthesis and cell growth. In humans, p27^kip1^ inhibits cyclin-dependent kinases (cdks) [42], which aid in promoting the transitions between cell-cycle phases. Overexpression of p27^kip1^ in human cells leads to cell-cycle arrest in the G1 phase, and when *foxo* and, subsequently, *p27*^*kip1*^, is upregulated [41,43]. Co-expression of *foxo* and constitutively active *Ras2*, which can induce G1/S progression and cell proliferation, is able to partially rescue the phenotype in the eye that is observed with an overexpression of *foxo* alone [7]. An increase in *foxo* activity appears to result in a decrease in cell proliferation. The double mutants, having both hypomorphic alleles of *Akt1* and null alleles of *foxo*, more closely resemble the *foxo* mutants. Analysis of ommatidia number shows an epistatic effect whereas an argument could be made in the comparison of ommatidia area for a slight synergistic enhancement of the phenotype. Regardless this suggests that without the presence of the *foxo* gene product, the hypomorphic alleles of *Akt1* do not cause the same reduction of growth and strongly suggests that *foxo* is necessary for the processes that lead to compensatory growth.

## Conclusions

Through the generation of clones, we were able to uncover a more severe effect of these *Akt1* hypomorphs upon the control of growth. Originally, the generation of somatic clones was utilized to study homozygous tissue in a heterozygous organism when the homozygotes themselves were not viable. However, when used to study homozygous tissues of viable, yet small and developmentally delayed organisms, this system can expose a subtle phenotype previously obscured by compensatory proliferation. Developmental delay is a common phenomenon associated with many genetic mutations and could potentially play a significant role in the final phenotype. Generation of somatic clones would eliminate this developmental timing factor, thereby clarifying the impact a genetic mutation has on cellular processes including growth.

### Animal ethics

This study was conducted under the approval of the Animal Care Committee of Memorial University of Newfoundland as a Category of Invasiveness Level A protocol under the project title of “Genetic, biochemical and molecular analysis of cell survival and cell death in *Drosophila melanogaster”* (protocol number: 14-09-BS).

## Abbreviations

foxo: Forkhead box subgroup “O”
Ilp: Insulin-like peptide
InR: Insulin receptor
OPE: Ommatidia per eye
